# Severe Acute Respiratory Syndrome Coronavirus 2 Transmission Potential, Iran, 2020

**DOI:** 10.3201/eid2608.200536

**Published:** 2020-08

**Authors:** Kamalich Muniz-Rodriguez, Isaac Chun-Hai Fung, Shayesteh R. Ferdosi, Sylvia K. Ofori, Yiseul Lee, Amna Tariq, Gerardo Chowell

**Affiliations:** Georgia Southern University, Statesboro, Georgia, USA (K. Muniz-Rodriguez, I.C.-H. Fung, S.K. Ofori);; The Translational Genomics Research Institute, Phoenix, Arizona, USA (S.R. Ferdosi);; Georgia State University School of Public Health, Atlanta, Georgia, USA (Y. Lee, A. Tariq, G. Chowell)

**Keywords:** 2019 novel coronavirus disease, coronavirus disease, COVID-19, severe acute respiratory syndrome coronavirus 2, SARS-CoV-2, viruses, respiratory infections, zoonoses, communicable diseases, epidemiology, infections, Iran

## Abstract

To determine the transmission potential of severe acute respiratory syndrome coronavirus 2 in Iran in 2020, we estimated the reproduction number as 4.4 (95% CI 3.9–4.9) by using a generalized growth model and 3.5 (95% CI 1.3–8.1) by using epidemic doubling time. The reproduction number decreased to 1.55 after social distancing interventions were implemented.

Since early 2020, Iran has been experiencing a devastating epidemic of coronavirus disease (COVID-19) (*1*). To determine the transmission potential of severe acute respiratory syndrome coronavirus 2 and thereby guide outbreak response efforts, we calculated basic reproduction numbers (R_0_). During the early transmission phase, R_0_ quantifies the average number of secondary cases generated by a primary case in a completely susceptible population, absent interventions or behavioral changes. R_0_>1 indicates the possibility of sustained transmission; R_0_<1 implies that transmission chains cannot sustain epidemic growth. As the epidemic continues, the effective reproduction number (R_e_) offers a time-dependent record of the average number of secondary cases per case as the number of susceptible persons becomes depleted and control interventions take effect. We used 2 methods to quantify the reproduction number by using the curve of reported COVID-19 cases in Iran and its 5 regions (Appendix Table 1). The Georgia Southern University Institutional Review Board made a non–human subjects determination for this project (H20364), under the G8 exemption category.

For method 1, we used a generalized growth model (*2*) with the growth rate and its scaling factor to characterize the daily reported incidence. Next, we simulated the calibrated generalized growth model by using a discretized probability distribution of the serial interval and assuming a Poisson error structure (Appendix).

We based method 2 on calculation of the epidemic’s doubling times, which correspond to the times when the cumulative incidence doubles and are estimated by using the curve of cumulative daily reported cases. To quantify parameter uncertainty, we used parametric bootstrapping with a Poisson error structure around the number of new reported cases to derive 95% CIs (*3**–**5*). Assuming exponential growth, the epidemic growth rate is equal to ln(2)/doubling time. Assuming that the preinfectious and infectious periods follow an exponential distribution, R_0_ ≈ (1 + growth rate × serial interval) (Appendix) (*6*).

For both methods, the serial interval was assumed to follow a gamma distribution; mean (± SD) = 4.41 (± 3.17) days (*7*; C. You et al., unpub. data, https://www.medrxiv.org/content/10.1101/2020.02.08.20021253v2). We used MATLAB version R2019b (https://www.mathworks.com) and R version 3.6.2 (https://www.r-project.org) for data analyses and creating figures. We determined a priori that α = 0.05.

Using Wikipedia as a starting point, we double-checked the daily reported new cases during February 19–March 19, 2020 (the day before the Iranian New Year) against official Iran press releases and other credible news sources and corrected the data according to official data (Appendix Tables 2, 3, Figure 1). Incident cases for the 5 regions were missing for 2 days (March 2–3), which we excluded from our analysis. Because the reported national number of new cases did not match the sum of new cases reported in Iran’s 5 regions on March 5, we treated each time series as independent and used the data as reported. Using method 1, we estimated R_0_ data for February 19–March 1, 2020. Using method 2, we estimated R_0_ from the early transmission phase (February 19–March 1, 2020) and R_e_ based on the growth rate estimated during March 6–19, 2020, when the epidemic slowed, probably reflecting the effect of social distancing.

Using method 1, we estimated an R_0_ of 4.4 (95% CI 3.9–4.9) for COVID-19 in Iran. We estimated a growth rate of 0.65 (95% CI 0.56–0.75) and a scaling parameter of 0.96 (95% CI 0.93–1.00) (Appendix Table 4). The scaling parameter indicated near-exponential epidemic growth (Figure). Using method 2, we found that during February 19–March 1, the cumulative incidence of confirmed cases in Iran had doubled 8 times. The estimated epidemic doubling time was 1.20 (95% CI 1.05–1.45) days, and the corresponding R_0_ estimate was 3.50 (95% CI 1.28–8.14). During March 6–19, the cumulative incidence of confirmed cases doubled 1 time; doubling time was 5.46 (95% CI 5.29–5.65) days. The corresponding R_e_ estimate was 1.55 (95% CI 1.06–2.57) (Appendix Table 5, Figures 7, 8). Our results are robust and consistent with Iran’s COVID-19 R_0_ estimates of 4.7 (A. Ahmadi et al., unpub. data, https://www.medrxiv.org/content/10.1101/2020.03.17.20037671v3) and 4.86 (E. Sahafizadeh, unpub. data, https://www.medrxiv.org/content/10.1101/2020.03.20.20038422v2) but higher than the R_0_ of 2.72 estimated by N. Ghaffarzadegan and H. Rahmandad (unpub. data, https://www.medrxiv.org/content/10.1101/2020.03.22.20040956v1).

**Figure:**
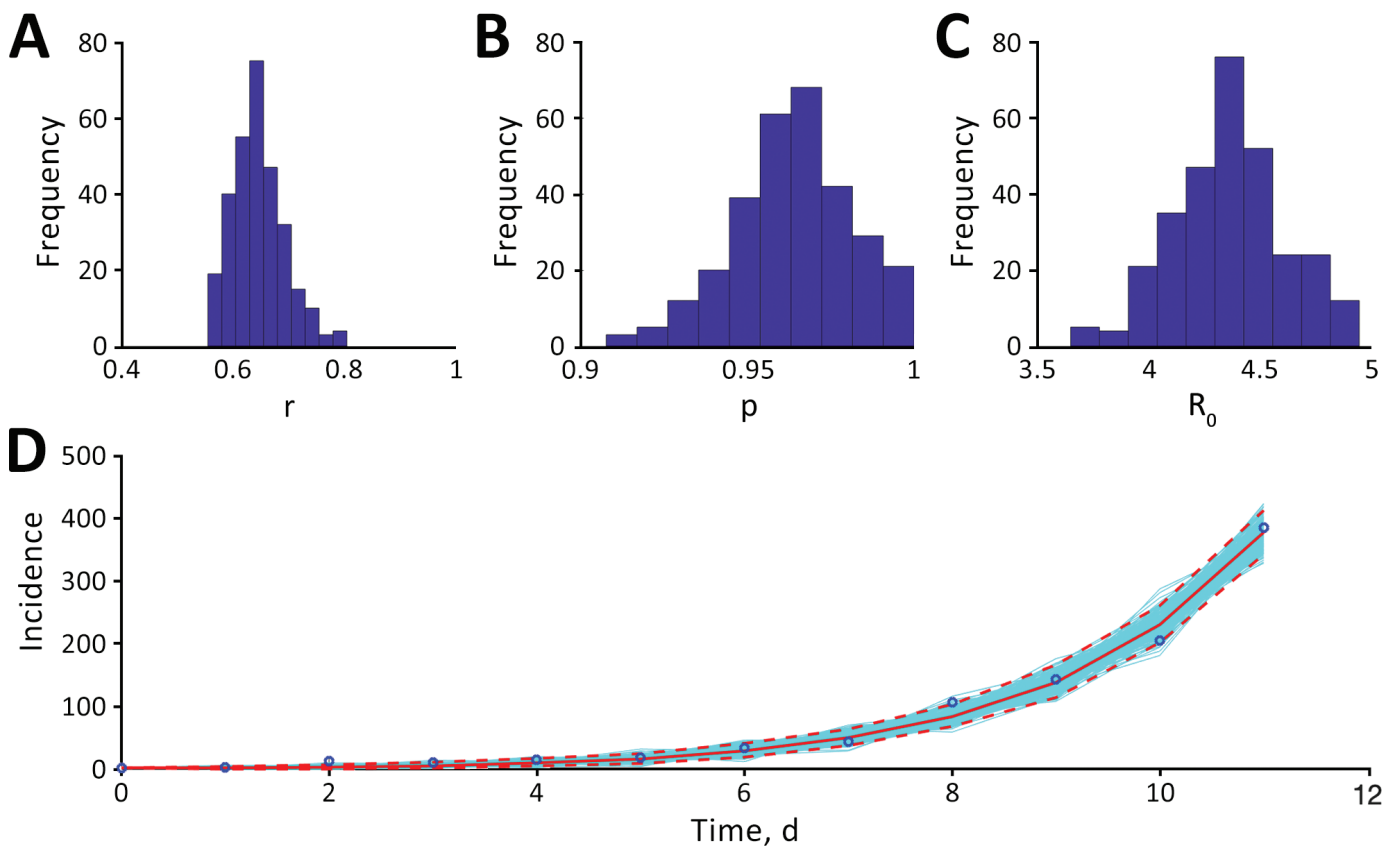
Estimates of transmission potential for severe acute respiratory syndrome coronavirus 2 in Iran, 2020. A) Growth rate, r; B) scaling of the growth rate parameter, p; C) mean basic reproduction number, R_0_; and D) fit of the generalized growth model (method 1) to the Iran data, assuming Poisson error structure as of March 1, 2020. Dashed lines indicate 95% CIs.

Our study has limitations. Our analysis is based on the number of daily reported cases, whereas it would be ideal to analyze case counts by dates of symptoms onset, which were not available. Case counts could be underreported because of underdiagnosis, given subclinical or asymptomatic cases or limited testing capacity to test persons with mild illness. The rapid increase in case counts might represent a belated realization of epidemic severity and rapid catching up and testing many persons with suspected cases. If the reporting ratio remains constant over the study period, and given the near-exponential growth of the epidemic’s trajectory, our estimates would remain reliable. Although data are not stratified according to imported and local cases, we assumed that persons were infected locally because transmission has probably been ongoing in Iran for some time (*8*).

Although the COVID-19 epidemic in Iran has slowed substantially, the situation remains dire. Tighter social distancing interventions are needed to bring this epidemic under control.

Appendix Supplementary methods and results for study of severe acute respiratory syndrome coronavirus transmission potential in Iran, 2020.

## References

[1] Wood G. Coronavirus could break Iranian society [cited 2020 Feb 29]. https://www.theatlantic.com/ideas/archive/2020/02/iran-cannot-handle-coronavirus/607150/

[2] Viboud C, Simonsen L, Chowell G. A generalized-growth model to characterize the early ascending phase of infectious disease outbreaks. Epidemics. 2016;15:27–37. 10.1016/j.epidem.2016.01.002 27266847 PMC4903879

[3] Banks HT, Hu S, Thompson WC. Modeling and inverse problems in the presence of uncertainty: CRC Press; 2014.

[4] Chowell G, Ammon CE, Hengartner NW, Hyman JM. Transmission dynamics of the great influenza pandemic of 1918 in Geneva, Switzerland: Assessing the effects of hypothetical interventions. J Theor Biol. 2006;241:193–204. 10.1016/j.jtbi.2005.11.026 16387331

[5] Chowell G, Shim E, Brauer F, Diaz-Dueñas P, Hyman JM, Castillo-Chavez C. Modelling the transmission dynamics of acute haemorrhagic conjunctivitis: application to the 2003 outbreak in Mexico. Stat Med. 2006;25:1840–57. 10.1002/sim.2352 16158395

[6] Vynnycky E, White RG. An introduction to infectious disease modelling. Oxford (UK): Oxford University Press; 2010.

[7] Nishiura H, Linton NM, Akhmetzhanov AR. Serial interval of novel coronavirus (COVID-19) infections. Int J Infect Dis. 2020;93:284–6. 10.1016/j.ijid.2020.02.060 32145466 PMC7128842

[8] Tuite AR, Bogoch II, Sherbo R, Watts A, Fisman D, Khan K. Estimation of coronavirus disease 2019 (COVID-19) burden and potential for international dissemination of infection from Iran. Ann Intern Med. 2020. 10.7326/M20-0696 PMC7081176 32176272

