# Supplementary material for: Severe Acute Respiratory Syndrome Coronavirus 2 Transmission Potential, Iran, 2020
Source: Emerg Infect Dis. 2020 Aug;26(8):1915–7. doi: 10.3201/eid2608.200536 (PMC7392448; doi:10.3201/eid2608.200536)
Supplement: Appendix — Supplementary methods and results for study of severe acute respiratory syndrome coronavirus transmission potential in Iran, 2020. [file 20-0536-Techapp-s1.pdf]

Article DOI: https://doi.org/10.3201/eid2608.200536

Severe Acute Respiratory Syndrome Coronavirus Transmission Potential, Iran, 2020

Appendix

Estimate of reproduction number from daily reported cases (Method 1)

Using the daily curve of reported cases in Iran, we estimate the reproduction number of COVID-19 (Appendix Table 2, Figure 1). For this purpose, we first characterize the daily reported incidence using the generalized growth model (GGM) (*1*). This model characterizes the growth profile via two parameters: the growth rate parameter (r) and the scaling of the growth rate parameter (p). The model captures diverse epidemic profiles ranging from constant incidence ($p = 0$), sub-exponential or polynomial growth ($0 < p < 1$), and exponential growth ($p = 1$) (*1*). The serial interval is assumed to follow a gamma distribution with a mean of 4.41 days and a standard deviation of 3.17 days based on recent reports ([*2*]; C. You et al., unpub. data, https://www.medrxiv.org/content/10.1101/2020.02.08.20021253v2).

Next, to estimate the most recent estimate of R_t, we simulate the progression of incident reported cases from the calibrated GGM, and apply the discretized probability distribution (ρ_i) of the serial interval using the renewal equation (*3–5*) given by

$$R_{t_i} = \frac{I_i}{\sum_{j=0}^{i}(I_{i-j})\rho_i}.$$

In the renewal equation we denote the total incidence at calendar time t_i by I_i. Here, the denominator represents the total number of cases that contribute to the incidence cases at time t_i. Next, we estimate R_t from 300 simulated curves assuming a Poisson error structure to derive the uncertainty bounds around our estimate of the reproduction number (*6*) (Appendix Table 4).

Estimate of reproduction number from epidemic doubling times (Method 2)

We ran simulation to generate 10,000 sets of estimates of epidemic doubling times for a given time series of cumulative number of reported cases (Appendix Table 3), and to compute the arithmetic mean of each set. Then, the harmonic mean of these estimates was calculated across 10,000 sets of simulations and was reported as our estimated epidemic doubling time, with 95% confidence interval (Appendix Table 5).

Next, we drew 10,000 random values for the serial interval from a gamma distribution with a mean of 4.41 days and a standard deviation of 3.17 days (*2*); C. You et al., unpub. data, https://www.medrxiv.org/content/10.1101/2020.02.08.20021253v2). We generate 10,000 values for the reproduction number by calculating the reproduction number for each pair of values (arithmetic mean of epidemic doubling time and serial interval respectively) following the equation as in Vynnycky and White (*7*), Table 4.1, Equation 4.14:

Reproduction number = 1 + growth rate × serial interval

We reported the mean and 95% confidence intervals (CI) of the 10,000 estimated values of the reproduction number (Appendix Table 5).

Results for Regions of Iran

Iran is geographically arranged into five regions, each of which contains a number of provinces (see Appendix Table 1). In addition to estimating the reproduction number of COVID-19 across Iran, we further analyzed the data for each of the five regions of Iran.

Appendix Tables 4 and 5, and Figures 2–8, present our estimates for Regions 1 to 5 using Methods 1 and 2. The estimates obtained from Method 1 had a smaller variance. The estimates obtained from Method 2 had a larger variance, given the large variance of the serial interval estimate that we used. Given that the 95% CIs of our reproduction number estimates obtained via Method 2 were large, they overlapped with those obtained via Method 1.

From February 19 through March 1, it appeared that whether it was for Iran as a whole, or for its five regions, the point estimate of the estimated basic reproduction number for each region was 2.0 or higher before the effect of social distancing interventions kicked in. From March 6 through 19, the effective reproduction number for each region had dropped to a range

from 1.48 of Region 3 to 1.77 of Region 5. However, the 95% CI of each region's estimate does not overlap with 1. Therefore, the transmission of SARS-CoV-2 (that causes COVID-19) remains active in all 5 regions of Iran (as effective reproduction number >1) before the Persian New Year Day of March 20, 2020.

This study in the context of recent pre-print literature on the 2020 COVID-19 epidemic in Iran

On April 2, 2020, we searched for "COVID-19" and "Iran" on medRXiv pre-print servers and identified 6 manuscripts that fit mathematical or statistical models to COVID-19 case count data obtained via official Iranian reports. See Appendix Table 6 for a summary. Three of these papers provide reproduction number estimates (A. Ahmadi et al., unpub. data, https://www.medrxiv.org/content/10.1101/2020.03.17.20037671v3; N. Ghaffarzadegan et al., unpub. data, ttps://www.medrxiv.org/content/10.1101/2020.03.22.20040956v1; Sahafizadeh et al., unpub. data, https://www.medrxiv.org/content/10.1101/2020.03.20.20038422v2). Our estimates of the basic reproduction number are comparable to theirs. These three papers also identified a lower effective reproduction number for the Iranian epidemic once social distancing interventions kicked in. However, only one paper suggested that the effective reproduction number has dropped below unity by March 20, 2020 (N. Ghaffarzadegan et al., unpub. data, ttps://www.medrxiv.org/content/10.1101/2020.03.22.20040956v1). The other two estimated an effective reproduction number >1 (A. Ahmadi et al., unpub. data, https://www.medrxiv.org/content/10.1101/2020.03.17.20037671v3; Sahafizadeh et al., unpub. data, https://www.medrxiv.org/content/10.1101/2020.03.20.20038422v2), that is consistent with our results.

References

1. Viboud C, Simonsen L, Chowell G. A generalized-growth model to characterize the early ascending phase of infectious disease outbreaks. Epidemics. 2016;15:27–37. PMID: 27266847

2. Nishiura H, Linton NM, Akhmetzhanov AR. Serial interval of novel coronavirus (COVID-19) infections. Int J Infect Dis. 2020;93:284–6. PubMed https://doi.org/10.1016/j.ijid.2020.02.060

3. Nishiura H, Chowell G. Early transmission dynamics of Ebola virus disease (EVD), West Africa, March to August 2014. Euro Surveill. 2014;19:20894. PubMed https://doi.org/10.2807/1560-7917.ES2014.19.36.20894

4. Nishiura H, Chowell G. The effective reproduction number as a prelude to statistical estimation of time-dependent epidemic trends. In: Chowell G, Hyman JM, Bettencourt LMA, Castillo-Chavez C, editors. Mathematical and Statistical Estimation Approaches in Epidemiology. 2009. p. 103–21.

5. Paine S, Mercer GN, Kelly PM, Bandaranayake D, Baker MG, Huang QS, et al. Transmissibility of 2009 pandemic influenza A(H1N1) in New Zealand: effective reproduction number and influence of age, ethnicity and importations. Euro Surveill. 2010;15:19591. PubMed

6. Chowell G. Fitting dynamic models to epidemic outbreaks with quantified uncertainty: a primer for parameter uncertainty, identifiability, and forecasts. Infect Dis Model. 2017;2:379–98. PubMed https://doi.org/10.1016/j.idm.2017.08.001

7. Vynnycky E, White RG. An introduction to infectious disease modelling. Oxford: Oxford University Press; 2010.

Appendix Table 1. Regions and provinces of Iran.

Region	Provinces
1	Qom Province (QOM), Tehran Province (TEH), Mazandaran Province (MAZ), Alborz Province (ALB), Semnan Province (SEM), Golestan Province (GOL), Qazvin Province (QAZ).
2	Esfahan (ESF), Fars (FRS), Hormozgan (HOR), Kohgiluyeh and Boyer-Ahmad (KOH), Chaharmahal and Bakhtiari (CHA), and Bushehr (BUS).
3	Gilan Province (GIL), Ardabil Province (ARD), East Azerbajian Province (AZS), West Azerbajian Province (AZG), Kurdistan (or Kordestan) Province (KUR).
4	Markazi (MAR), Hamedan (HAM), Khazistan (KHZ), Kermanshah (KRS), Lorestan (LOR), and Ilam (ILM).
5	Razavi Khorasan (KHR), Sistan and Baluchestan (SIS), Yazd (YAZ), South Khorasan (KHS), Kerman (KER), and North Khorasan (KHN).

Appendix Table 2. Daily number of new reported COVID-19 cases in Iran and its five regions*

Date (yyyy-mm-dd)	Iran	Region 1	Region 2	Region 3	Region 4	Region 5
2020-02-19	2	2	-	-	-	-
2020-02-20	3	2	-	-	1	-
2020-02-21	13	11	-	2	-	-
2020-02-22	10	10	-	-	-	-
2020-02-23	15	12	-	2	1	-
2020-02-24	18	11	2	2	3	-
2020-02-25	34	29	2	2	0	1
2020-02-26	44	21	5	10	6	2
2020-02-27	106	58	8	32	6	2
2020-02-28	143	98	10	32	3	0
2020-02-29	205	125	20	31	23	6
2020-03-01	385	245	23	28	67	22
2020-03-02	523	422.1805†	20.9259†	30.8184†	42.1267†	11.1281†
2020-03-03	835	528.6418†	17.0741†	39.4146†	59.4522†	16.0099†
2020-03-04	586	424	15	54	62	31
2020-03-05	591‡	256	150	122	49	69
2020-03-06	1234	693	180	175	112	74
2020-03-07	1076	663	126	99	82	106
2020-03-08	743	378	104	68	140	53
2020-03-09	595	265	89	119	92	30
2020-03-10	881	508	72	63	138	100
2020-03-11	958	485	200	90	78	105
2020-03-12	1075	605	62	150	183	75
2020-03-13	1289	614	147	237	121	170
2020-03-14	1365	624	173	272	186	110
2020-03-15	1209	562	149	156	126	216
2020-03-16	1053	480	189	160	97	127
2020-03-17	1178	566	118	203	134	157
2020-03-18	1192	505	255	199	120	113
2020-03-19	1046	359	160	210	140	177

*COVID-19, coronavirus disease.
†The daily new cases for each of the 5 regions on March 2 and 3, 2020, were interpolated using cubic spline. ‡The reported number of the national total on March 5 does not match the sum of the 5 regions.

Appendix Table 3. Daily cumulative number of daily reported COVID-19 cases in Iran*

Date (yyyy-mm-dd)	Iran	Region 1	Region 2	Region 3	Region 4	Region 5
2020-02-19	2	2	-	-	-	-
2020-02-20	5	4	-	-	1	-
2020-02-21	18	15	-	2	1	-
2020-02-22	28	25	-	2	1	-
2020-02-23	43	37	-	4	2	-
2020-02-24	61	48	2	6	5	-
2020-02-25	95	77	4	8	5	1
2020-02-26	139	98	9	18	11	3
2020-02-27	245	156	17	50	17	5
2020-02-28	388	254	27	82	20	5
2020-02-29	593	379	47	113	43	11
2020-03-01	978	624	70	141	110	33
2020-03-02	1501	NR	NR	NR	NR	NR
2020-03-03	2336	NR	NR	NR	NR	NR
2020-03-04	2922	1984	183	345	273	82
2020-03-05	3513	2240	333	467	322	151
2020-03-06	4747	2933	513	642	434	225
2020-03-07	5823	3596	639	741	516	331
2020-03-08	6566	3974	743	809	656	384
2020-03-09	7161	4239	832	928	748	414
2020-03-10	8042	4747	904	991	886	514
2020-03-11	9000	5232	1104	1081	964	619
2020-03-12	10075	5837	1166	1231	1147	694
2020-03-13	11364	6451	1313	1468	1268	864
2020-03-14	12729	7075	1486	1740	1454	974
2020-03-15	13938	7637	1635	1896	1580	1190
2020-03-16	14991	8117	1824	2056	1677	1317
2020-03-17	16169	8683	1942	2259	1811	1474
2020-03-18	17361	9188	2197	2458	1931	1587
2020-03-19	18407	9547	2357	2668	2071	1764

***NR, not reported by the Iranian Government.**

Appendix Table 4. Method 1 (February 19 through March 1, 2020): Estimated epidemic growth rate (95% Confidence intervals, CI), scaling parameter (95% CI) and basic reproduction number obtained via a generalized growth model.

**Location**	Epidemic growth rate (r, 95% CI)	Scaling parameter, p	Basic reproduction number (95% CI)
Iran	0.65 (0.56, 0.75)	0.96 (0.93, 1)	4.4 (3.9, 4.9)
Region 1	0.55 (0.51, 0.65)	0.99 (0.94, 1)	4.3 (3.8, 4.6)
Region 2	1.20 (0.58, 2.20)	0.76 (0.52, 1)	3.4 (2.3, 5.0)
Region 3	3.00 (1.50, 5.00)	0.52 (0.37, 0.69)	2.1 (1.7, 2.7)
Region 4	0.75 (0.67, 0.96)	0.97 (0.87, 1)	5.8 (4.4, 6.4)
Region 5	0.83 (0.68, 1.30)	0.94 (0.72, 1)	6.0 (3.8, 7.3)

Appendix Table 5. Method 2 (February 19 through March 1, and March 6 through 19, 2020): Estimated epidemic doubling time (95% Confidence intervals, CI), epidemic growth rate (95% CI) and the basic (or effective) reproduction number (95% CI) obtained via Method 2. Epidemic growth rate (*r*) = ln(2)/doubling time. Reproduction number was calculated based on equation: R0 = 1 + growth rate × serial interval, assuming serial interval following a gamma distribution with a mean of 4.41 days and a standard deviation of 3.17 days.

	Estimated epidemic doubling times (95% CI)*		Epidemic growth rate (r, 95% CI)		Basic Reproduction number (95% CI) §	Effective Reproduction number (95% CI) §
**Location**	**Feb 19 – Mar 1**	**Mar 6 – 19**	**Feb 19 – Mar 1**	**Mar 6 – 19**	**Feb 19 – Mar 1**	**Mar 6 – 19**
Iran	1.20 (1.05, 1.45)	5.46 (5.29, 5.65)	0.58 (0.48, 0.66)	0.13 (0.12, 0.13)	3.50 (1.28, 8.14)	1.55 (1.06, 2.57)
Region 1	1.32 (1.16, 1.61)	6.04 (5.80, 6.29)	0.53 (0.43, 0.60)	0.11 (0.11, 0.12)	3.28 (1.26, 7.59)	1.50 (1.06, 2.42)
Region 2	1.12 (0.87, 1.59)	5.71 (5.32, 6.04)	0.62 (0.44, 0.80)	0.12 (0.11, 0.13)	3.69 (1.29, 8.84)	1.53 (1.06, 2.50)
Region 3	1.38 (1.12, 1.78)	6.23 (5.86, 6.51)	0.50 (0.39, 0.62)	0.11 (0.11, 0.12)	3.17 (1.24, 7.24)	1.48 (1.06, 2.37)
Region 4	1.67 (1.43, 2.30)	5.19 (4.58, 5.79)	0.41 (0.30, 0.49)	0.13 (0.12, 0.15)	2.79 (1.20, 6.15)	1.58 (1.07, 2.65)
Region 5	1.13 (0.92, 1.61)	3.92 (3.54, 4.32)	0.62 (0.43, 0.75)	0.18 (0.16, 0.20)	3.66 (1.30, 8.79)	1.77 (1.09, 3.19)

*Harmonic mean of the arithmetic means of 10,000 sets of simulated epidemic doubling times.

§Calculated based on the 10,000 arithmetic means of 10000 sets of simulated epidemic doubling times and 10,000 random numbers drawn from a gamma distribution with a mean serial interval of 4.41 days (SD: 3.17 days).

Appendix Table 6. Manuscript pre-prints on the COVID-19 epidemic in Iran fit to Iranian official reports available on medRXiv as of April 2, 2020*

Papers	medRXiv pre-print version as of Apr 2, 2020	Iranian official COVID-19 reports†: time range	Other data sources	Methods	Basic reproduction number	Effective reproduction number after social distancing implemented
A. Ahmadi et al., unpub. data, https://www.medrxiv.org/content/10.1101/2020.03.17.20037671v3	Pre-print version 2	February 19 through March 19, 2020	Not applied	Logistic differential equation, Gompertz differential equation, Von Bertalanffy's differential growth equation, Cubic Polynomial Polynomials	4.7	1.75 (Gompertz)
N. Ghaffarzadegan et al., unpub. data, https://www.medrxiv.org/content/10.1101/2020.03.22.20040956v1	Pre-print version 1	February 19 through March 19, 2020	Unofficial reports; and international media news reports	Compartmental (SEIR) model fit to reported data	2.72 (90% CI, 2.57, 2.92)	<1 by March 20, 2020
E. Sahafizadeh et al., unpub. data, https://www.medrxiv.org/content/10.1101/2020.03.20.20038422v2	Pre-print version 2	February 22 through March 18, 2020	Not applied	Compartmental (SIR) model fit to data	4.86 (1st week, with Feb 19, 2020 as day zero)	4.5 (2nd week); 4.29 (3rd week); 2.1 (4th week)
A. Zahiri et al., unpub data, https://www.medrxiv.org/content/10.1101/2020.03.29.20046532v1	Pre-print version 1	February 19 through March 24, 2020	China's official COVID-19 reports	Compartmental (SIR) model fit to data; also fit Iranian data to the epidemic curve in China	Not reported	Not reported
B. Zareie et al., unpub data, https://www.medrxiv.org/content/10.1101/2020.03.19.20038950v1	Pre-print version 1	January 22‡ to March 8, 2020	China's official COVID-19 reports	Compartmental (SIR) model fit to China's data to obtain parameter estimates and then applied to Iran's data	Not reported	Not reported
C. Zhan et al., unpub data, https://www.medrxiv.org/content/10.1101/2020.03.08.20032847v1	Pre-print version 1	February 19 through March 6, 2020	China's official COVID-19 reports	Compartmental (SEIR) model fit to epidemic data in 367 cities in China to generate historical profiles of epidemics (sets of parameter values) that were compared to observed data in South Korea, Italy and Iran to make predictions based on the best-fit epidemic profiles of the SEIR model	Not reported	Not reported

*Notes: Manuscript pre-prints uploaded to medRXiv server (medrxiv.org) are working papers and they are not (or have not yet been) peer-reviewed.
†The abstract of Zareie et al. mentioned "January 22"; however, the first confirmed cases of Iran were reported on February 19, 2020.
‡Official daily reports of deaths, recovered and cumulative number of infected individuals from the Iranian Ministry of Health and Medical Education.

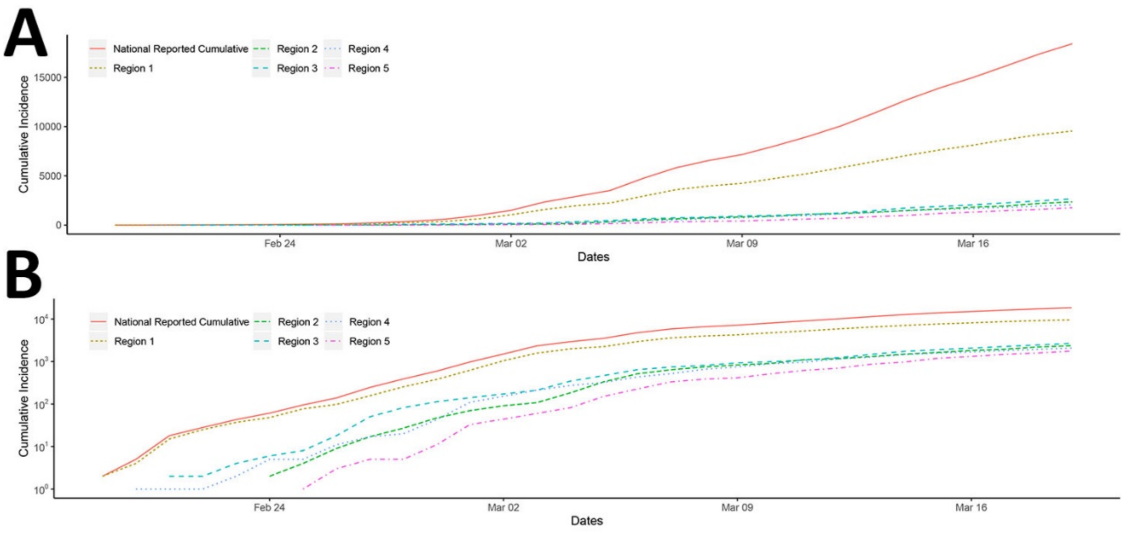

Appendix Figure 1. Cumulative incidence curve of Iran and its Regions 1 to 5. The data for the five regions on March 2 and 3, 2020 were imputed.

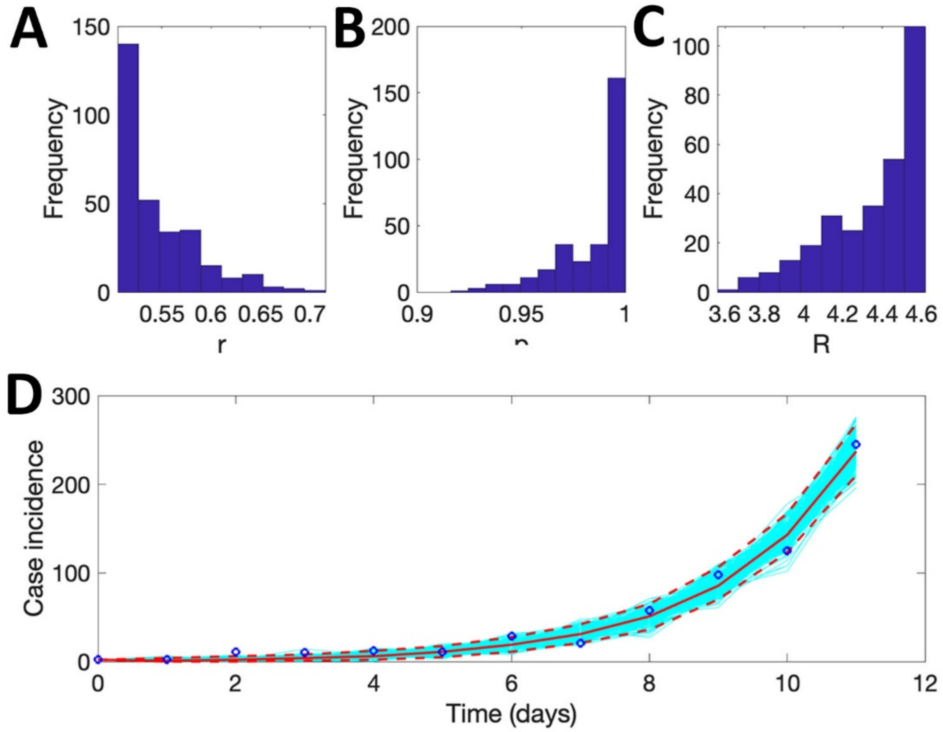

Appendix Figure 2. Method 1: Region 1. The mean basic reproduction number of COVID-19 in Region 1, Iran, with 95% confidence interval. Estimates for growth rate, r, and the scaling of the growth rate parameter, p, are also provided. The plot in the lower panel depicts the fit of the Generalized Growth Model to the Iranian data assuming Poisson error structure as of March 1, 2020.

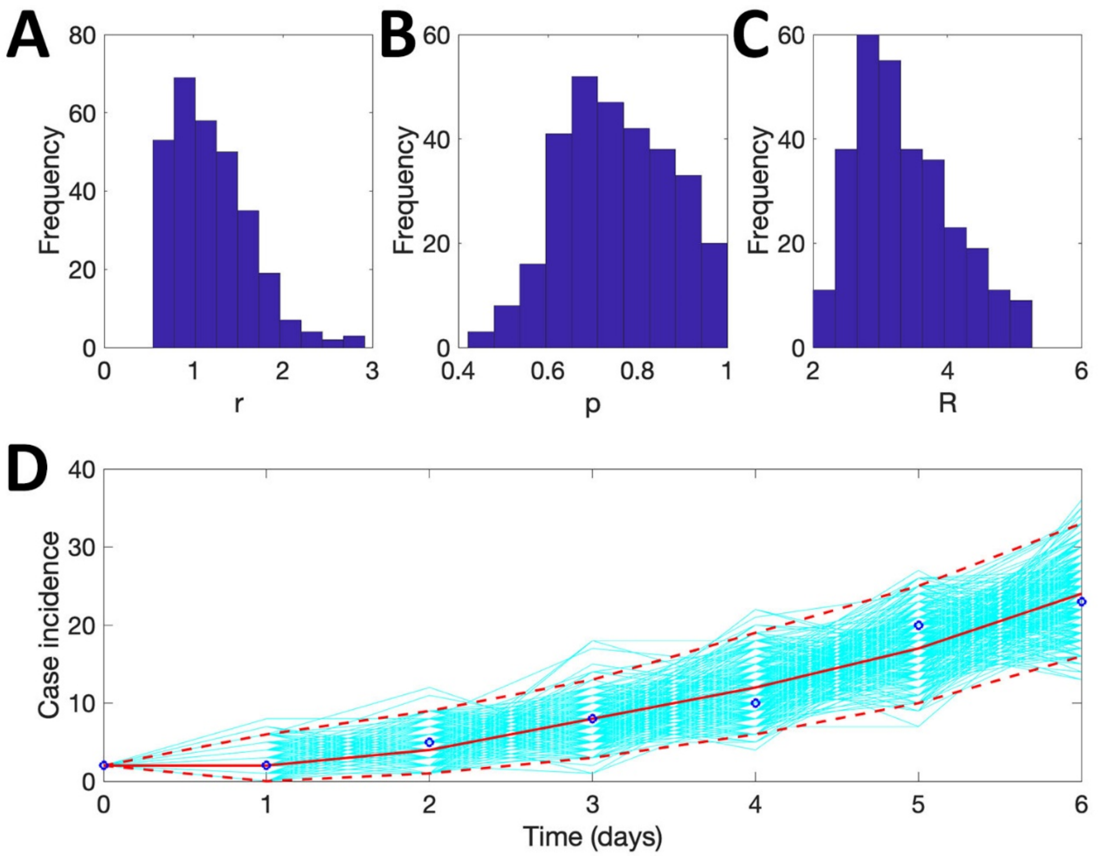

Appendix Figure 3. Method 1: Region 2. The mean basic reproduction number of COVID-19 in Region 2, Iran, with 95% confidence interval. Estimates for growth rate, r, and the scaling of the growth rate parameter, p, are also provided. The plot in the lower panel depicts the fit of the Generalized Growth Model to the Iranian data assuming Poisson error structure as of March 1, 2020.

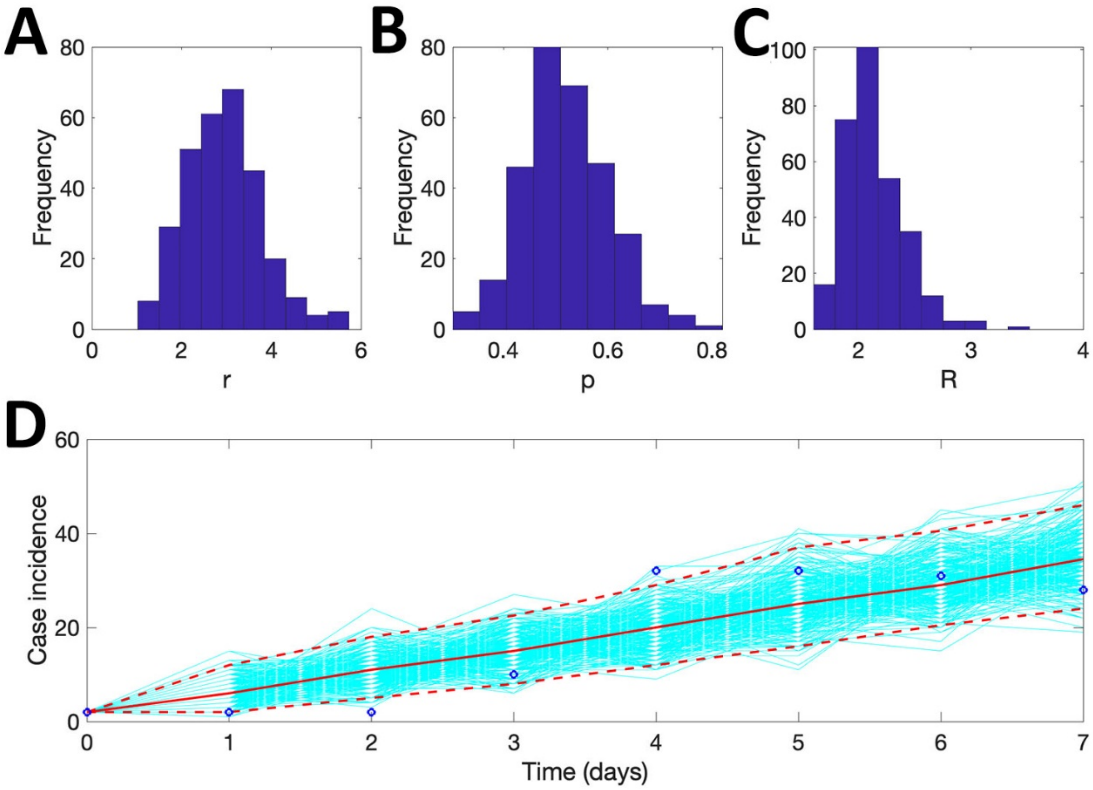

Appendix Figure 4. Method 1: Region 3. The mean basic reproduction number of COVID-19 in Region 3, Iran, with 95% confidence interval. Estimates for growth rate, r, and the scaling of the growth rate parameter, p, are also provided. The plot in the lower panel depicts the fit of the Generalized Growth Model to the Iranian data assuming Poisson error structure as of March 1, 2020.

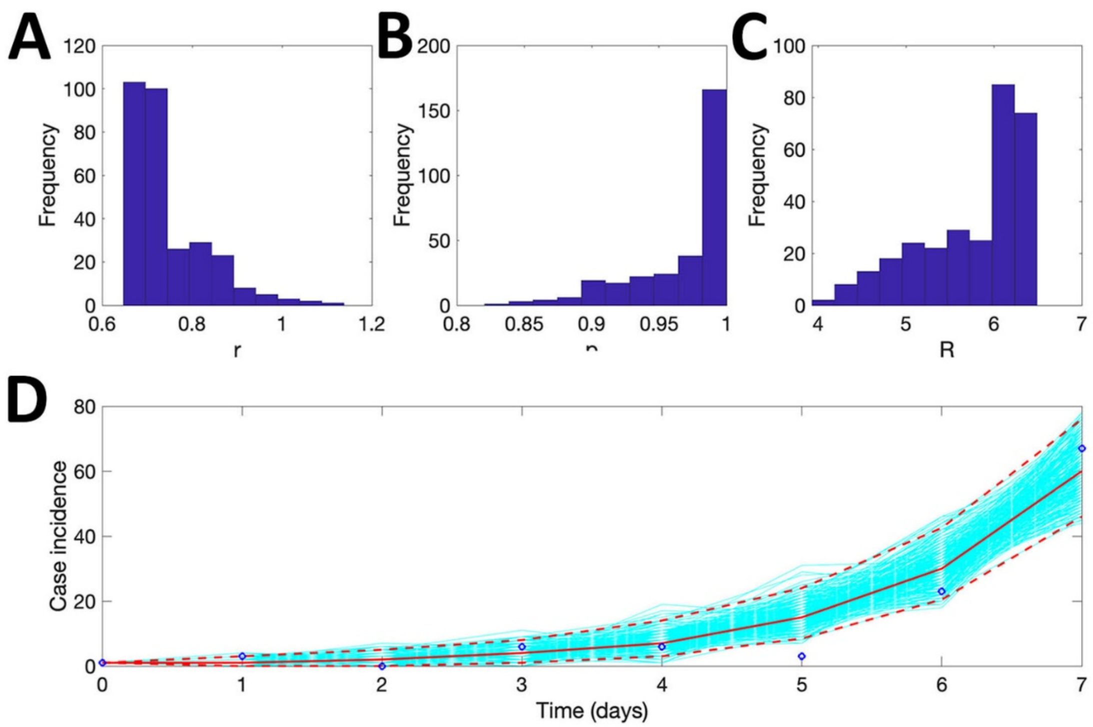

Appendix Figure 5. Method 1: Region 4. The mean basic reproduction number of COVID-19 in Region 4, Iran, with 95% confidence interval. Estimates for growth rate, r, and the scaling of the growth rate parameter, p, are also provided. The plot in the lower panel depicts the fit of the Generalized Growth Model to the Iranian data assuming Poisson error structure as of March 1, 2020.

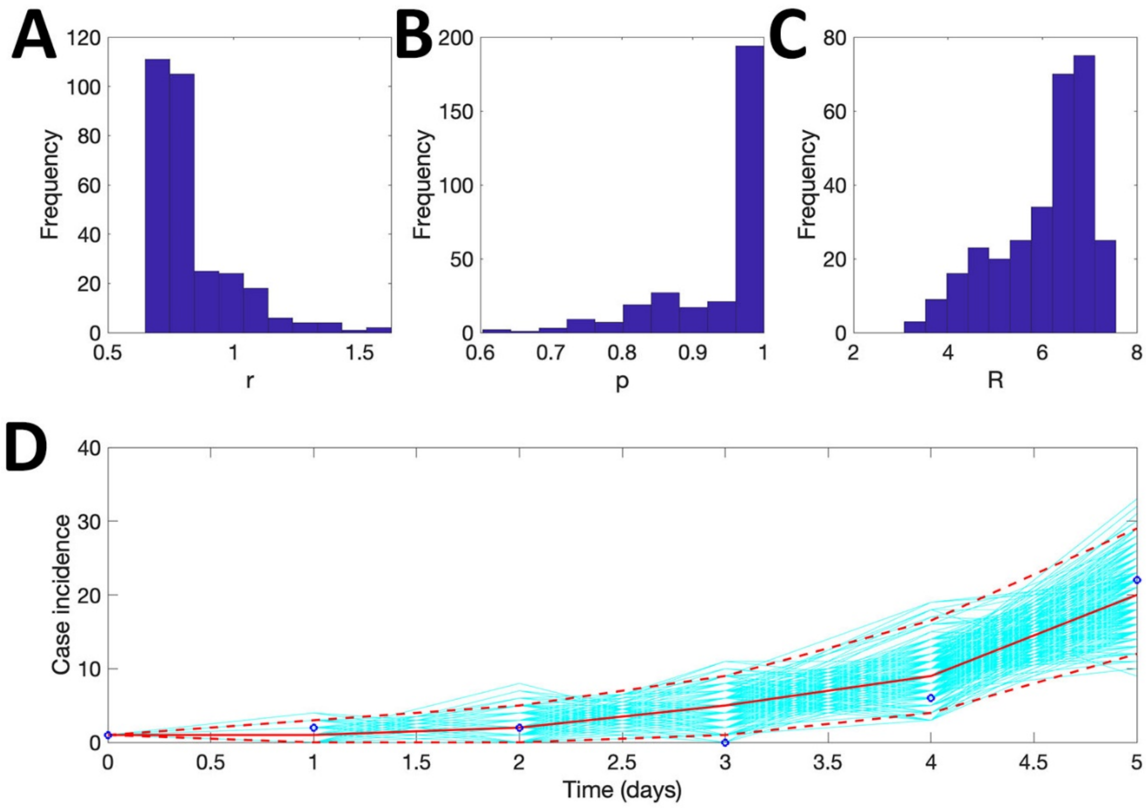

Appendix Figure 6. Method 1: Region 5. The mean basic reproduction number of COVID-19 in Region 5, Iran, with 95% confidence interval. Estimates for growth rate, r, and the scaling of the growth rate parameter, p, are also provided. The plot in the lower panel depicts the fit of the Generalized Growth Model to the Iranian data assuming Poisson error structure as of March 1, 2020.

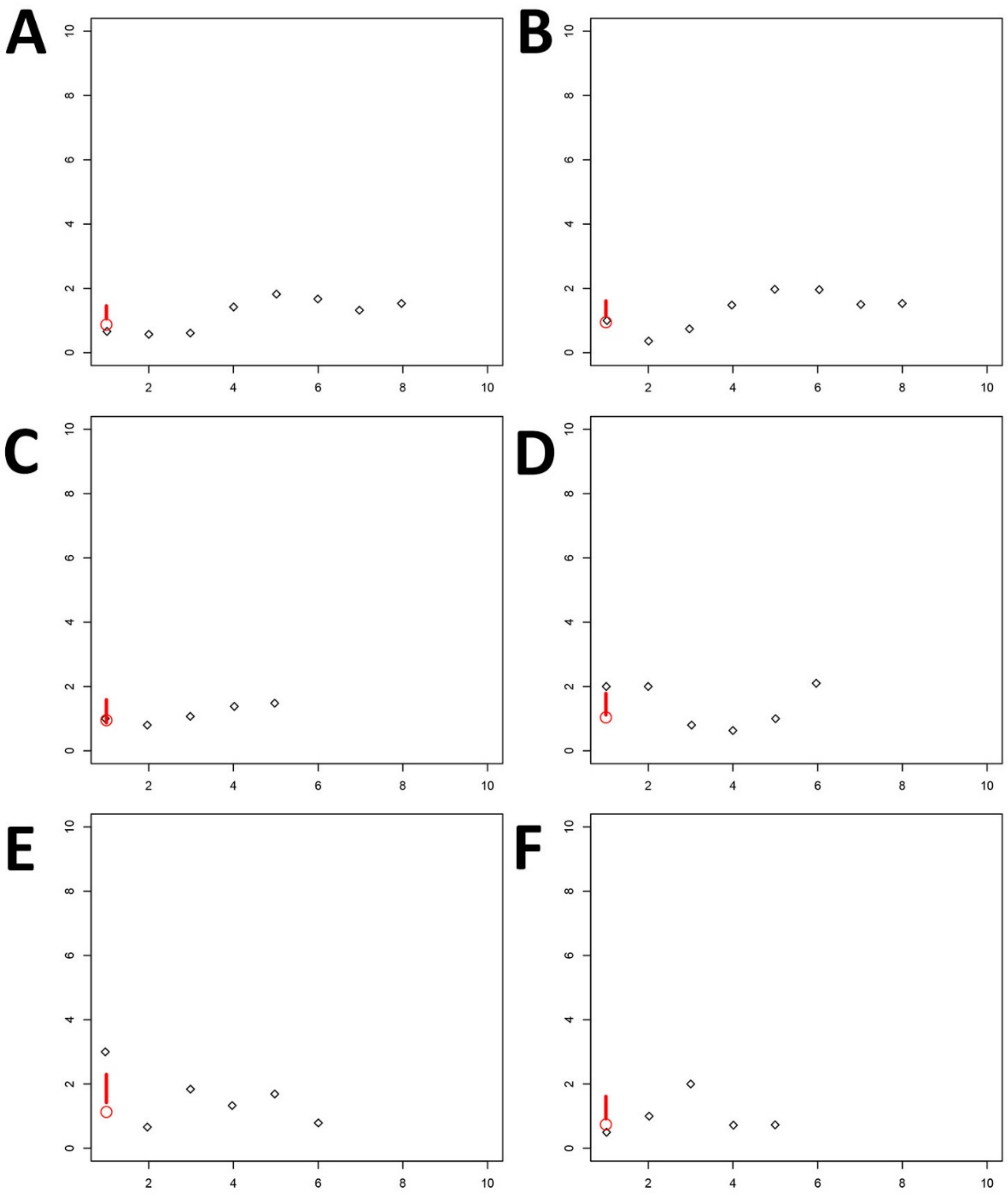

Appendix Figure 7. Method 2: Part 1 (February 19 through March 1, 2020): The COVID-19 epidemic doubling time (black diamond) and the harmonic mean of the arithmetic means of 10,000 estimates with 95% confidence intervals (circle and bar) of Iran and its five regions.

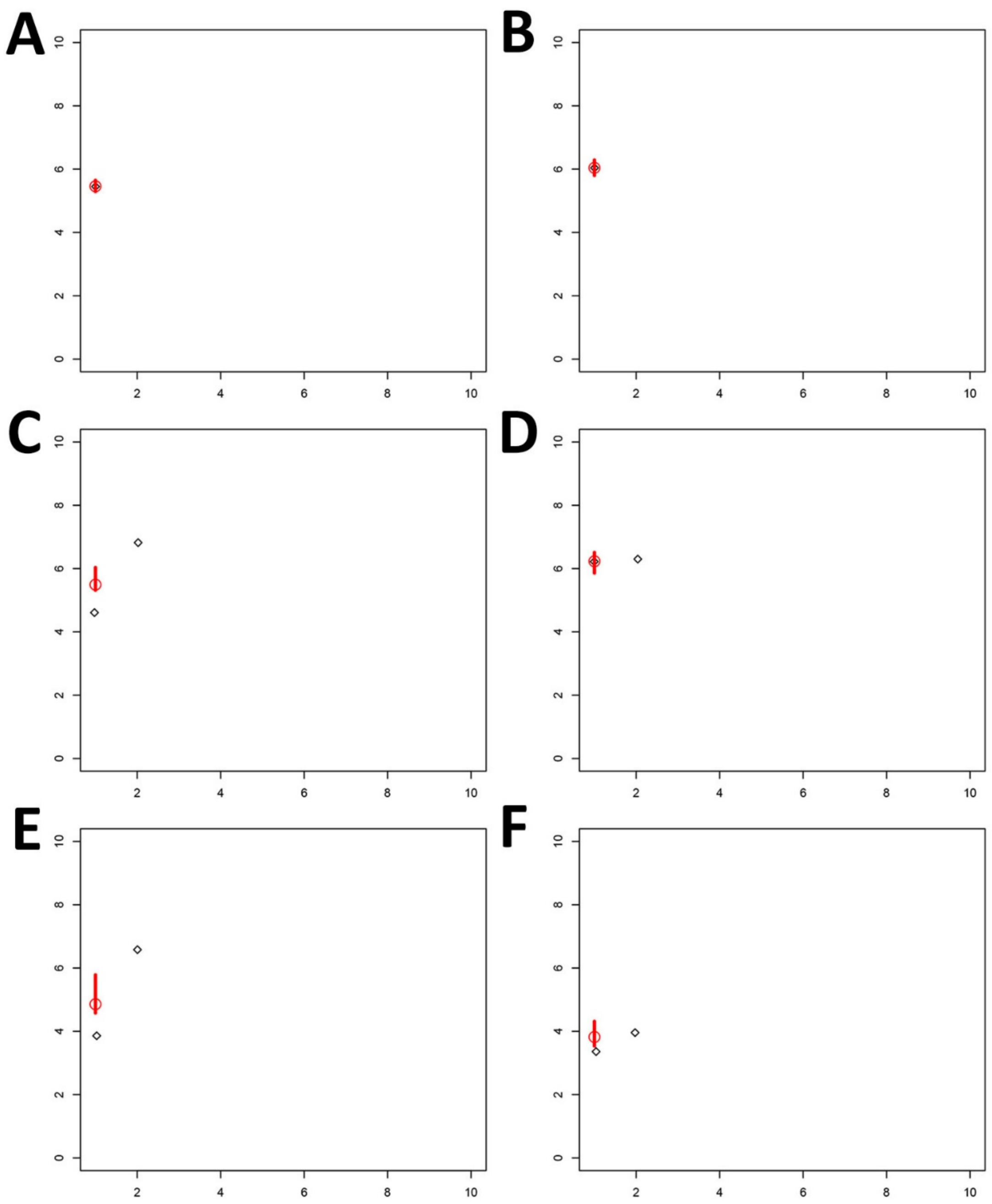

Appendix Figure 8. Method 2: Part 2 (March 6 through 19, 2020): The COVID-19 epidemic doubling time (black diamond) and the harmonic mean of the arithmetic means of 10,000 estimates with 95% confidence intervals (circle and bar) of Iran and its five regions.
